# Analysis of deviation from classical $d_0^2$-law for biochar conversion in an oxygen-enriched and temperature-controlled environment

**DOI:** 10.1038/s41598-022-22910-w

**Published:** 2022-11-01

**Authors:** N. Mohammed Asheruddin, Anand M. Shivapuji, Srinivasaiah Dasappa

**Affiliations:** Centre for Sustainable Technologies, Indian Institute of Science, Bangalore, 560012 India

**Keywords:** Chemical engineering, Energy, Renewable energy, Chemical engineering

## Abstract

Combustion of char has conventionally been reported to be diffusion controlled. Analytically, the process is reported to follow second order initial diametric ($d_0$) dependence $(d_0^\beta ; \beta =2)$ for both single-film (no CO combustion) and two-film models (CO burns in a concentric sphere over the particle). However, experimental investigations indicate deviation from classical diffusion limit with $\beta$ exceeding 2.00 and going as high as 2.37. Videography investigations depict luminous film engulfing the particle for certain Temperature-Oxygen concentration-Particle diameter combinations (for which, $\beta \ge 2$). The observed deviation is hypothesized to convective resistance offered by the *CO* generated on the surface to motion of $CO_2$ towards the surface. This results in reduced $CO_2$ concentration at the surface with enhanced conversion time being the implication (hence, $\beta >2$). Such convective resistance remains unaccounted for in the prevailing analytical models. The *CO* dominated film thickness is enhanced with temperature and reactant concentration, increasing the convective resistance, and further deviating from $d_0^2$ behaviour. The analytical solution shows that in presence of a convectively expanding *CO* film, total conversion time is a function of film diameter while also being dependent on $d_0^2$. The hypothesis is validated by comparing analytical estimates with experimentally observed film diameter and conversion time.

## Introduction

Combustion of solid fuels has been established as a diffusion-limited process and a well-defined analytical approach leads to the classical $d_0^2$ law for the particle conversion time^1^, where, $d_0$ corresponds to the initial particle diameter. In the case of char conversion in an oxidizing media, the $d_0^2$ law is reported to remain valid for the single film model (flameless) and two film (concentric CO flame) model^2^. It has also been explicitly established that under certain thermo-physical conditions (like temperature (T), reactant concentration $(X_{O_2 }$), diffusivity, tortuosity, heterogeneous reaction rates etc.), the conversion process can deviate from being diffusion-controlled, resulting in the initial diametric dependence (power of $d_0$) dropping below 2^3,4^. The limiting condition would be a linear initial diametric dependence $(d_0^1)$ for the case of purely kinetically controlled conversion process^1^. Basically, in the correlation $d_0^\beta$ the value of $\beta$ will be between 1 (kinetically limited) and 2 (diffusionally limited), inclusive of the extremes^5^.

Several researchers^1,6–11^ have analytically approximated limiting conditions for combustion of solid fuels under a range of conditions. However, no literature reports or addresses the circumvention of diffusion limit ($\beta = 2$). This is primarily due to extremely limited investigation in the high temperature oxygen enriched reactant regime^12,13^. The high temperature-high oxygen concentration environments are typically observed in oxy-fuel ($O_2-CO _2$ and $O_2-H_2O$) gasification process^14^. $O_2/CO_2$ ratios upto 1.05^15^ and $O_2/H_2O$ ratio of upto 2.2^16^ are typically reported. $O_2$ mole fractions in excess of 60% and the resulting high temperatures in practical oxyfuel gasification systems set the backdrop for the current study.

Towards investigating the initial diametric dependence on the char combustion process in oxygen enriched gaseous mixture, experimental investigations spanning a range of sizes, temperature and oxidizer concentration (oxygen fraction in oxygen-nitrogen mixture) have been carried out. This study focuses on the high temperature and high Oxygen concentration regime for milli-meter scale particles (8–20 mm). Experimental investigations indicate very interesting and unique observations on the diametric dependence with time for complete conversion. It is observed that beyond certain T and $X_{O_2}$, the power of $d_0$ surpasses the classical diffusion limit of 2. As a typical example, Table 1 presents the curve-fit for temperature and concentration parametric analysis, depicting $\beta$ value surpassing 2. The experimental results indicate a very clear and explicit boundary that separates the conventional diffusion controlled regime $(\beta \le 2)$ from the regime where the diametric dependence limit exceeds the power of 2. The trends and observation in the current work are unique and are being reported for the first time.

**Table 1:** Experimental results quantifying $t_c=\alpha d_0^\beta$ (a) varying T with 100% $O_2$, (b) varying $X_{O_2}$ at 1073 K.

T (K)	$\alpha$	$\beta$	$X_{O_2}$ (%)	$\alpha$	$\beta$
300	3.57	1.84	100	0.14	2.37
473	2.11	1.92	80	0.35	2.32
673	0.65	2.16	60	0.58	2.27
873	0.35	2.29	40	1.33	2.12
1073	0.14	2.37	20	5.94	1.82

The current article presents a detailed experimental investigation and analysis that addresses the observed deviation from the conventionally established initial diametric dependency. A hypothesis is proposed to address the observed deviation and the hypothesis is extended to an analytical solution by invoking the Sherwood number. The conversion time correlation as proposed by S. Turns^17^ and Annamalai et al.^1^ which is a function of initial diameter alone is extended by introducing a geometric scaling factor. The scaling factor is itself a function of T and $X_{O_2}$. The correlation accommodates the observed diametric dependence enabling the surpassing of the conventional limit, $\beta = 2$. The correlations are validated with experimental conversion times and it is observed that for a majority of cases the estimated conversion time remains within 10% of the measured conversion time with the maximum deviation limited to 25%.

## Methodology

### Experimental setup and procedure

**Figure 1:**
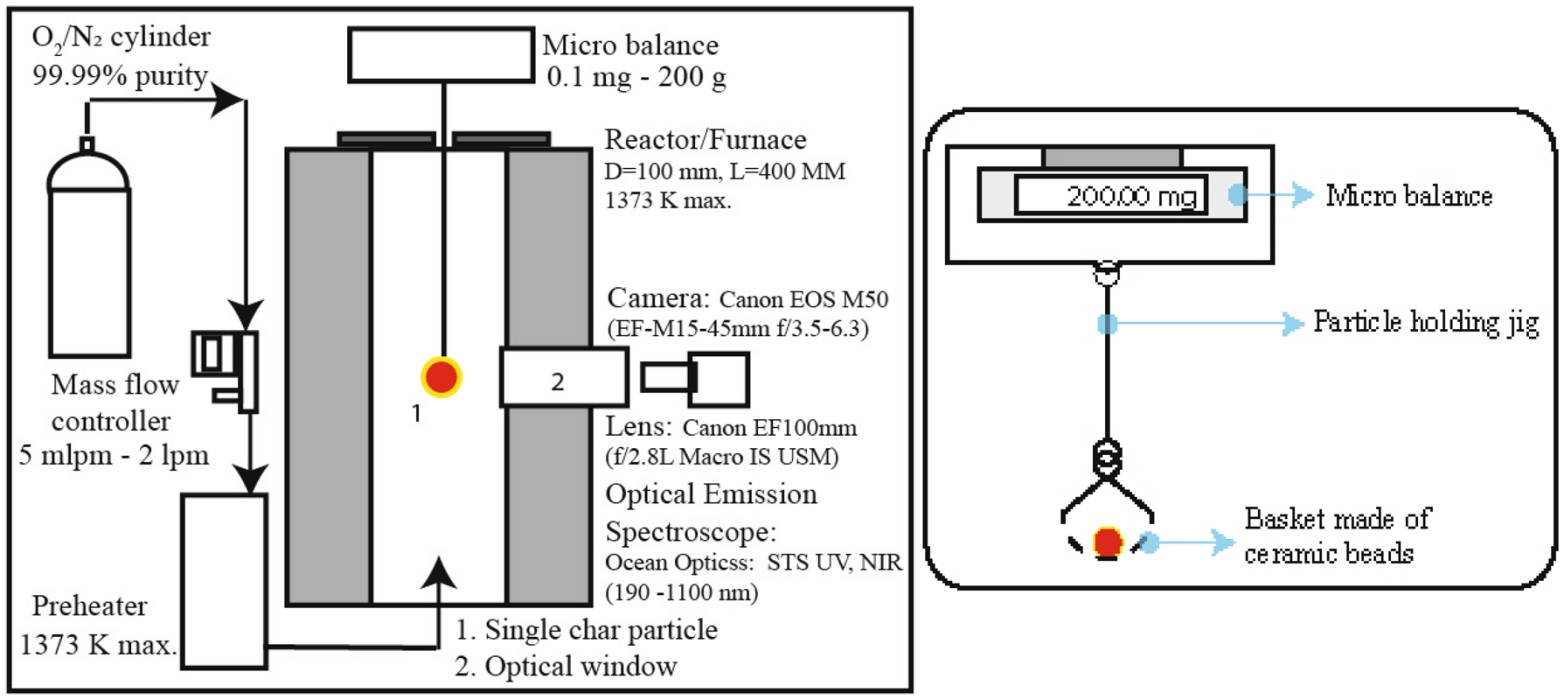
Single particle experimental setup and detailed illustration of particle holding mechanism.

The single char particle combustion experiments are carried out in a novel single-particle experimental facility engineered at the lab. The setup as shown in Fig. 1 consists of a high-temperature furnace with a ceramic reactor (100 mm diameter, 400 mm length) with an optical access. The char particle is suspended from a precision (100 $\mu$g least count) balance fixed at an elevated frame. The furnace body has one degree of freedom in the vertical direction while the furnace top supporting the weighing balance and the particle remains stationary. This arrangement enables introduction of the particle in the furnace only after desired steady state condition is achieved. The particle is freely suspended from the micro-balance with a particle-holding jig, as shown in Fig. 1. The particle-holding jig rests on the pan of micro-balance on one end and holds the ceramic bead basket on the other. The ceramic bead basket comprises four SS-310 strands laden with ceramic beads. The ceramic beads withstand the high temperature resulting from char combustion and limit conductive heat transfer from the particle (the thermal conductivity of ceramic is almost five times lower than steel). The reactant gas from ultra-high pure gas cylinders is regulated through an electronic mass flow controller and preheated to the experimental temperature in a dedicated heater and administered into the reactor. Towards achieving a flowrate which provides the necessary oxygen concentration at the particle vicinity, but not leading to convective effects (near-quiescent environment), particle Reynolds Number (Re) of 0.1 is estimated. In defining Re, particle diameter is considered and the thermophysical properties are estimated based on the experimental temperatures and reactant gas composition. The Re is used to calculate the oxidizer velocity; which is used to arrive at the oxidizer mass flowrate.

The experimental setup is contained in vibration resistant transparent enclosure to prevent any external factors interfering with the measurement. Also, prior to each experimental run, it was ensured that the furnace temperature, oxygen flowrate and temperature are stable at the set values. The particle diameter was measured in three orthogonal directions to ensure sphericity and visually checked to be free from structural deformities.

The experiments involve subjecting a single spherical char particles of initial diameter ($d_0$) to predetermined temperature and reactant concentration. The weight loss of the particle is continuously monitored using a sensitive weighing balance. Concurrently, through the optical window the particle combustion is observed, and captured through a digital single-lens reflex camera coupled with a macro lens. The macro lens facilitates extreme close-up of the particle combustion without loss of resolution. In a repeat experiment, the camera is replaced with an optical emission spectrometer (OES) for qualitative characterization of specie distribution around the particle. The key outputs from the experiments are the temporal evolution of mass, particle and flame diameter, total burn time, and specie distribution.

The experiments are carried out over a wide range of conditions as in Table 2. The temperature (T, K) indicated in Table 2 and further in the analysis corresponds to the furnace temperature. It is to be noted that the particle surface progressively reduces with combustion, and accurately measuring the surface temperature is instrumentally unfeasible. As such, furnace temperature is used as a reference, and this consideration has no implications on the inferences of the current work. It is also to be noted that at low furnace temperatures ($< 673 K$) there is no auto-ignition, and a pilot flame is used to ignite the particle. Post ignition the particle is allowed to burn in the desired ambient temperature. The effect of ignition is negligible as it is observed for less than 10% of the total combustion time^3,4^.

**Table 2:** Range of experimental conditions.

Parameter	Range
$d_0$ (mm)	8, 10, 12, 16, 18, 20
T (K)	300, 373, 473, 673, 873, 1073
$X_{O_2}$ (%, bal. $N_2$)	20, 40, 60, 80, 100

#### Sample preparation

The spherical char test sample are prepared by slow pyrolysis (300 K to 1073 K at 10 K min$^{-1}$; held at 1073 K for 60 min) of Beech wood (*Fagus sylvatica*) spheres under an inert ($N_2$) flux. The resulting char spheres are characterized (Table 3) for its composition, morphology diameter ($d_0$) and mass.

**Table 3:** Properties of char samples.

Property	Beech char	Property	Beech char
C (%)	76.4 ± 3.6	H (%)	3.4 ± 0.3
O (%)	19.6 ± 2.8	N (%)	0.6 ± 0.08
S (%)	0.01 ± 0.005		
Fixed carbon (%)	97.9 ± 1.1	Ash (%)	2.1 ± 0.7
Density (kg m$^{-3}$)	219.3 ± 31.1	Porosity	0.88 ± 0.02
BET surface area (${\rm m}^2\,{\rm g}^{-1}$)	254		

$^{\rm a}$ Proximate analysis of bone dry char as per ASTM Standards: ASTM E872, E1534.

$^{\rm b}$ Porosity is calculated based on non-porous carbon density.

### Image processing and analysis

**Figure 2:**
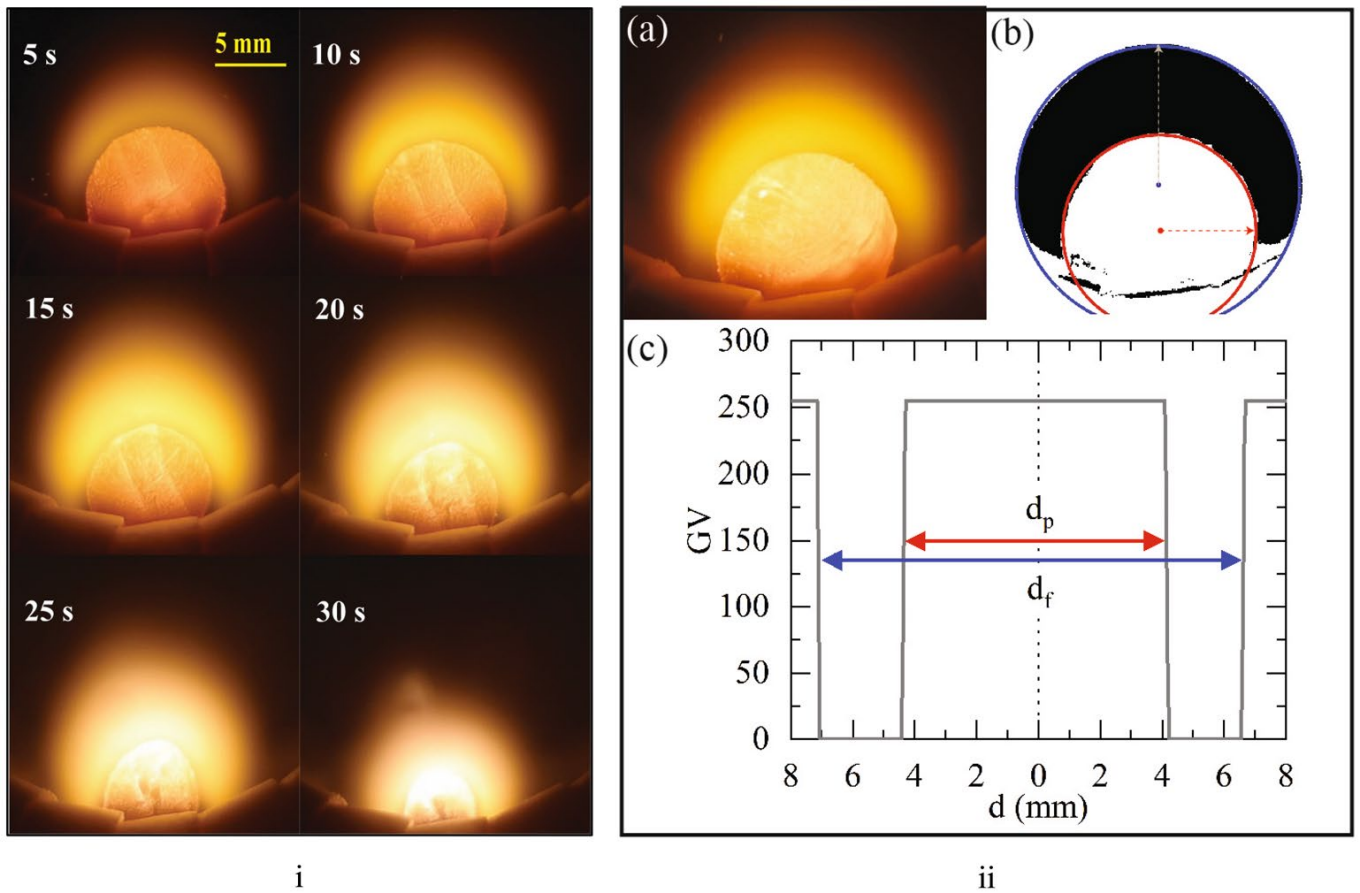
(**i**) Timelapse of a 10 mm diameter char particle subjected to reactive experiments of 1073 K and 100% $O_2$; (**ii-a**) Raw image, (**ii-b**) Processed image with circles fit to isolate the particle (red) and film (blue), (**ii-c**) measurements of particle diameter ($d_p$) and film diameter ($d_f$) in the processed image (Gray value (GV) profile across diameter (d)).

The particle combustion process is video recorded at 25 frames per second and individual frames are extracted for further processing. The particle is observed to progressively reduce in size with the combustion process with no visible cracking or fragmentation. A time-lapse of the combustion process of a 10 mm diameter particle subjected to 1073 K and 100% $O_2$, spaced five seconds apart is shown in Fig. 2. In Figure 2, the particle is held on a specially designed particle holding jig made of ceramic beads. The ceramic beads withstand the high temperature generated by the oxygen-enriched combustion in high temperature ambient. In addition, the relatively low thermal conductivity of ceramic significantly reduces heat loss from particle surface. The measurements and by extension the analysis has no influence of the ceramic beads as more than 90% of the particle surface is clearly visible at all times.

Towards quantifying the particle diameter and film diameter the raw images (Fig. 2ii-a) are subjected to Gaussian filter to eliminate any noise grains. The image is then segmented using color threshold to isolate the film based on the HSB (Hue-Saturation-Brightness) information of each pixel. The segmented image is further subjected to a black and white threshold which highlights the film alone as a black region (Gray value, GV=0) on a white background (GV=255) (Fig. 2ii-b). The outer boundary of the identified film region is the film-ambient interface, and the inner boundary is the film-particle interface. The offset of the film and particle centers is attributed to buoyancy effects. A scale is set to the images based on the known particle diameter at time, t = 0. The adopted approach is well established in the analysis of diffusion flame structure^18^.

In regards to the uncertainty in diameter measurement, with the resolution of each pixel being 50 microns, that would be the maximum error. A gray value (GV) profile across the radius (r) (Fig. 2ii-c) indicates the particle diameter and film diameter.

## Results and discussion

This section presents the experimental results (each data point presented has a minimum of 5 repeats) describing the nature of combustion followed by the presentation of hypothesis explaining the observed deviation from the classical $d^2$ law. The hypothesis is extended to the first principles through the introduction of Sherwood number and an analytical correlation is arrived at. The section culminates with validation of the established correlation.

### Temporal evolution of particle mass and diameter

**Figure 3:**
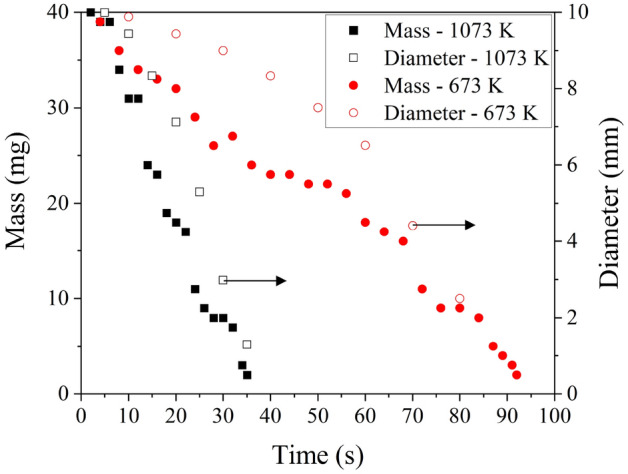
Evolution of mass (primary axis) and diameter (secondary axis) of a 10 mm diameter particle burning in 100% $O_2$ at 673 K and 1073 K.

The mass of particle and the diameter of the particle progressively reduce with time. Considering the 2% ash present in the char sample, upto 98% mass loss is tracked. With regards to the particle diameter, reduction in diameter till 90% particle conversion is mapped as beyond this point the particle disintegrates or converts asymmetrically. The evolution of mass and diameter of a 10 mm diameter particle burning in 100% $O_2$ at 673 K and 1073 K is shown in Fig. 3.

To check the sphericity of the particle during the conversion process, the conversion process is frozen at 25%, 50% and 75% of conversion (based on mass loss) by quenching and cooling the particles with $N_2$ flux at 0.01 $kg\, m^2\, s^{-1}$. Having frozen the conversion, the particles are retrieved, and the diameter is measured in the three orthogonal directions. It is found that the standard deviation in the diameter measurement in three orthogonal directions is nominal (of the order $\sim$ 0.1 mm). This negligible standard deviation indicates that the particle remains near-spherical throughout the conversion process. Further, noting that the process is diffusion-limited and the reactions occur at the particle’s surface, the surface area estimated with diameter measured from videography is compared with the surface area calculated from diameters in three orthogonal directions. It is found that the maximum difference in surface area is 1.3%. Conclusively, the particles are considered to behave like perfect spheres throughout the conversion process, with a diameter equivalent to the average of orthogonal measurements made from the videography.

### Regression analysis and diametric dependence

Tracking the reduction in diameter (*d*) of a particle from its initial size ($d_0$ at time, $t=0$) till its complete combustion ($t=t_c$) enables analyzing the temporal evolution of particle geometry. Adherence to the $d_0^2$ law requires a linear trend for a plot mapping $d^2$ against time. Similarly, the mass (*m*) of particle is tracked with time. For a spherical particle, in terms of mass, a linear trend in $d^2-t$ profile translates to a linearity in $m^{2/3}-t$ profile.

**Figure 4:**
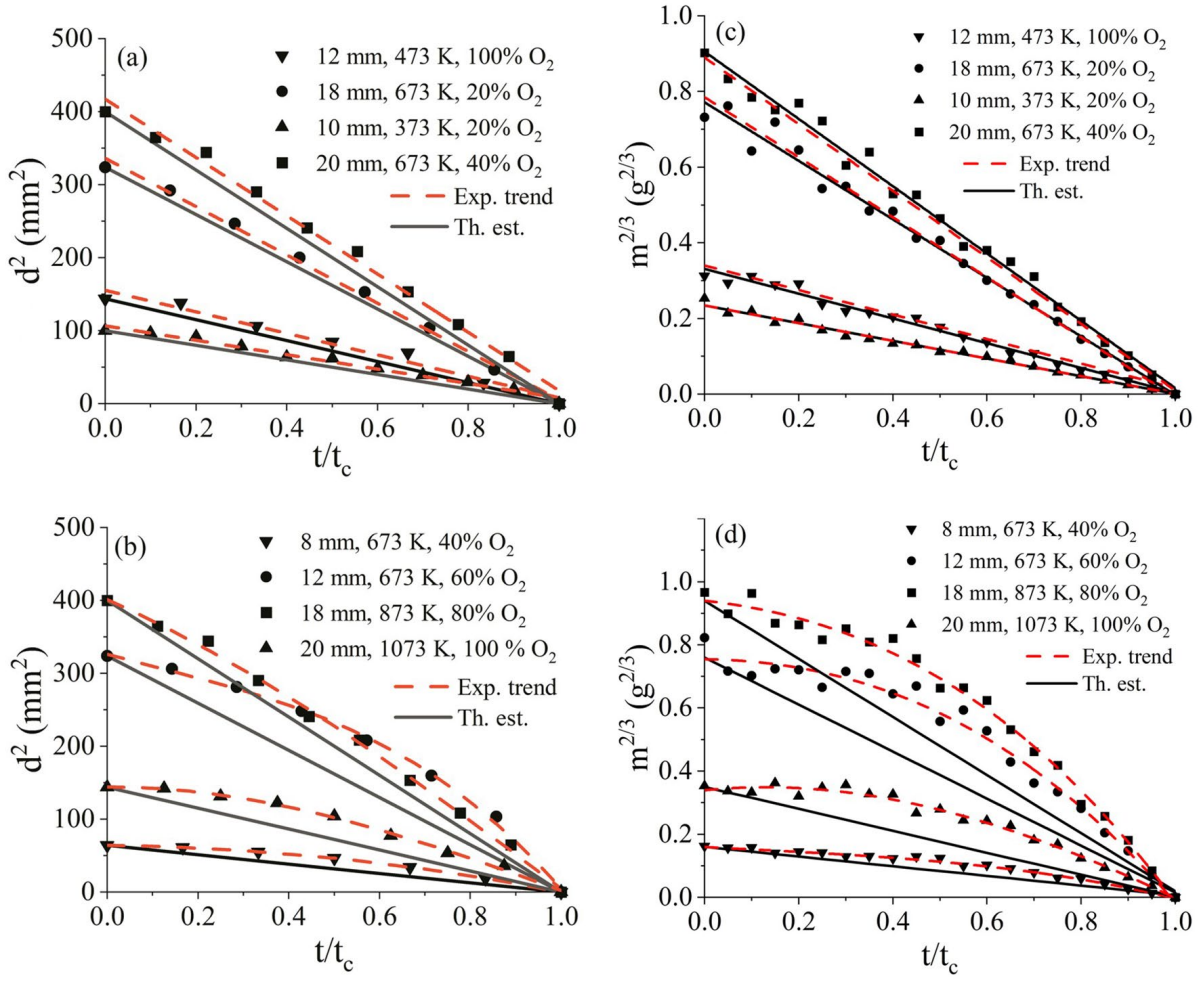
Temporal evolution of squared particle diameter ($d^2$) and particle mass ($m^{2/3}$) with normalized time ($t/t_c$) for different sized particles, temperature and $O_2$ concentrations (**a**) adherence to $d_0^2$ behavior ((**c**) corresponding mass loss profile), (**b**) deviating from $d_0^2$ behavior ((**d**) corresponding mass loss profile). (error in analyzing $d_p$ is 0.05 mm).

Figure 4 represents the evolution of diameter ($d^2$) and mass ($m^{2/3}$) against time with Fig. 4a and c identifying cases following the classical $d^2$ law, and Fig. 4b and d identifying the cases deviating the classical $d^2$ law (based on deviation of the experimental profile from linearity). In Fig. 4, the markers indicate the experimental data points, dashed lines indicate the experimental trends and continuous lines indicate the theoretical estimates (Ref. Eq. 5). The observed deviation is discussed in a subsequent section. While Fig. 4 presents the mass loss and diametric evolution for 8 experimental cases as representation, the discussion and understanding were found to be same across the full range (180 cases) of experimental data set.

It is observed that at $T < 473 K$ and $X_{O_2}$ $\le$ 20%, the conversion behavior is nearly linear. For the other conditions (T> 473 K, $X_{O_2}$ > 20%), the $d^2$-t and $m^{2/3}$-t profiles are observed to deviate from linearity across the range of particle sizes. A similar deviation in linearity of $d^2$-t profile is also observed in the experimental results of Kreitzberg et al.^7^, however the deviation is not quantified or discussed therein.

The identified conditions (T, $X_{O_2}$, $d_0$) under which $d_0^2-law$ remains valid and conditions leading to deviation from $d_0^2-law$ are consolidated as in Table 4. Under the “*Follows *$d_0^2-law$” and “*Deviates from* $d_0^2-law$” regions, all the particle sizes follow/deviate the $d_0^2-law$. However, in the intermediate region (T: 673–873 K; $X_{O_2}$: 40–80%), the validity of $d_0^2-law$ is dependent on particle size, specifically indicated in Table 4.

**Table 4:**
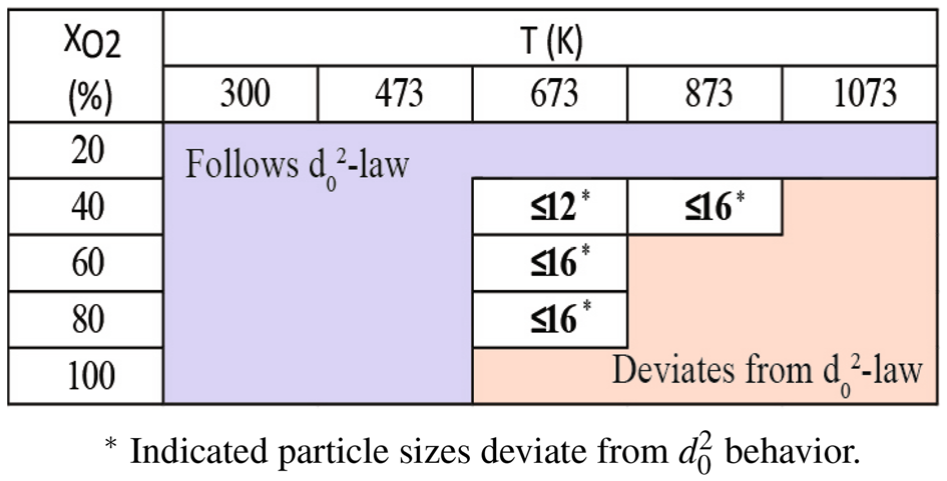
Conversion behavior for combination of diameter, temperature and $O_2$ concentration.

In reference to Table 4, while particles of all sizes (8–20 mm) adhere to $$d_0^2-law$$ at low-temperature, low-concentration conditions and particles of all sizes deviates from $$d_0^2-law$$ ($$d_0^2-d_0^{2.36})$$ at high-temperature, high-concentration conditions. There is a particular transition (marked in bold) where size dependence is evident. Smaller sized particles tend to deviate from $$d_0^2-law$$ while larger sized particles tend to adhere to $$d_0^2-law$$ for a particular thermophysical conditions. As an example, at 673 K and 60% $$O_2$$ smaller particle sizes ($$\le$$ 16 mm) deviate from $$d_0^2-law$$ due to higher surface temperatures, but at the same thermophysical condition, larger particle adhere to $$d_0^2-law$$ due to lower surface temperatures.

The interesting behavior of smaller particles having higher surface temperature is owing to the dynamics between heat generated by reactions and heat lost by radiation. The reaction rate for oxidation of spherical char particle in diffusion limited regime is established in the literature by various researchers^2,17^ as,

1 $$\begin{aligned} Burn \, rate=Sh\pi {\rho }_g{\mathscr {D}}d_p\mathrm {ln}\mathrm {}(1+B) \end{aligned}$$

It is noted from Eq. (1) that, $Burn rate \propto d_p$. Radiation from spherical particle can be formulated as,

2 $$\begin{aligned} Q_{rad}=A \epsilon \sigma (T_s^4-T_\infty ^4 )=\pi d_p^2 \epsilon \sigma (T_s^4-T_\infty ^4 ) \end{aligned}$$

It is noted from Eq. (2) that, $Q_{rad} \propto d_p^2$. Basically, the burn (reaction) rate of char particle is proportional to $d_p$, whereas the radiation loss is proportional to $d_p^2$. The implication being, the surface temperature for larger particle would be lesser than that of smaller particle due to higher radiative loss in larger particle.

The phenomena of smaller particles having higher surface temperature is experimentally observed and reported by Avedesian and Davidson^19^ in fluidised bed gasification. Annamalai and Caton^20^ establish the phenomena theoretically and report that, at 10% $O_2$ concentration the surface temperature of a 1 mm particle is 25 $^0C$ higher than that of 1.5 mm particle.

### Nature of $d_0$ dependence on conversion time

**Figure 5:**
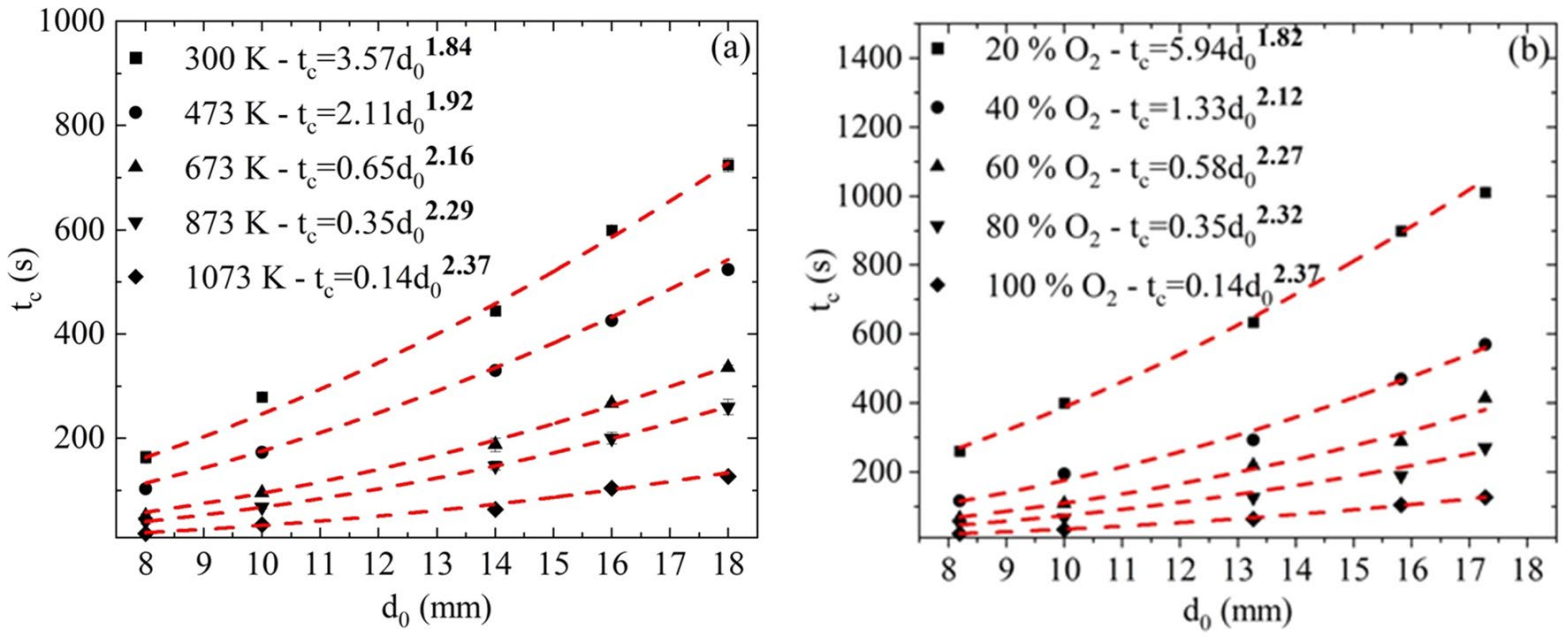
Dependence of initial particle diameter ($d_0$) on the complete conversion time ($t_c$) (a) for different temperatures and 100% $O_2$, (b) varying $O_2$ concentrations at 1073 K.

While the previous section established the deviation from classical $d^2$ law, the $\alpha$ and $\beta$ in the $t_c=\alpha d_0^ \beta$ correlation are quantified in this section. The total conversion time for each particle is mapped to its initial size resulting in a $t_c$ v/s $d_0$ plot for a particular T-$X_{O_2}$ combination. A power-law curve-fit to the plot quantifies $\alpha$ and $\beta$ in the correlation $t_c=\alpha d_0^ \beta$. This analysis is extended over the entire range of $T-X_{O_2}$ pairs presented in Table 2. The $t_c-d_0$ profiles for the full range of analysis are shown in Fig. 5, with the quantified $t_c=\alpha d_0^ \beta$ correlation (corresponding to goodness of fit of over 99%) presented as inset data. Figure 5a highlights the influence of temperature ($X_{O_2}$ constant at 100%) on the conversion process and the $t_c=\alpha d_0^ \beta$ correlation. Similarly, Fig. 5b indicates the influence of oxygen concentration (temperature held constant at 1073 K) on the conversion process.

Reviewing Fig. 5a and b, it is evident that, as the temperature increases the power of $d_0$ increases. $\beta \le 2$ is observed upto a temperature of 473 K (diffusion dominated regime) it surpasses the classical analysis limit of $\beta =2$ going as high as $\beta =2.37$ at 1073 K. Similar trend is observed for reactant concentration greater than 20% $O_2$. Additional analysis of the $\beta =2$ correlations in Fig. 5a and b indicates that while $\beta$ increases with an increase in T and $X_{O_2}$, a commensurate decrease in $\alpha$ is observed. For an increase in T from 300 K to 1073 K, while $\beta$ increases by upto 28%, the reduction in $\alpha$ is nearly twenty times. Similarly, increase in T-$X_{O_2}$ from 20% to 100% leads to 30% increase in $\beta$ and 42-times reduction in $\alpha$.

**Figure 6:**
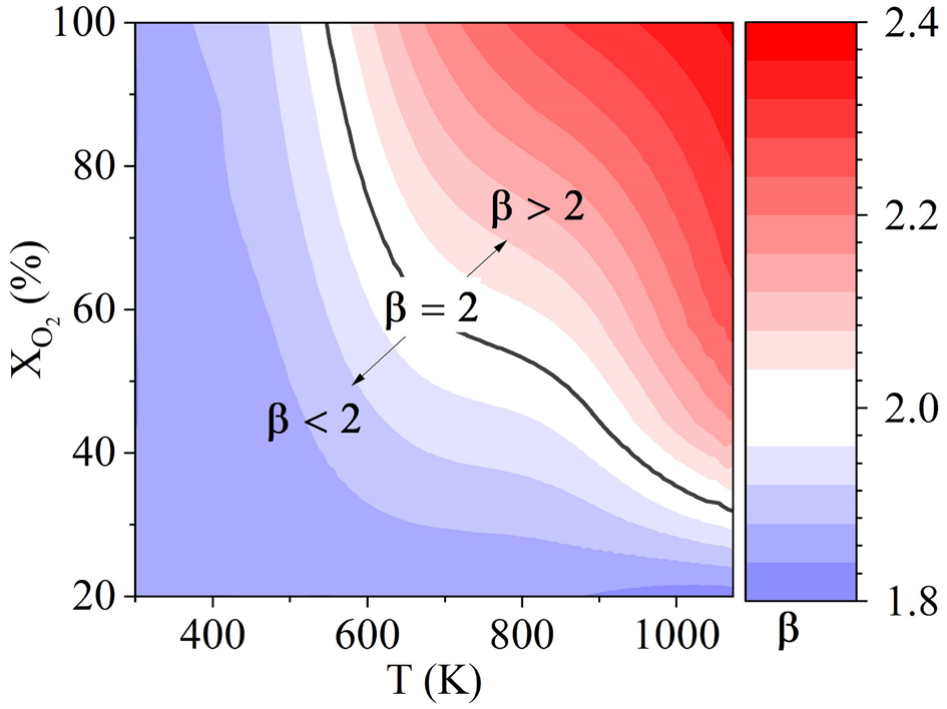
Variation of $\beta$ over a temperature range of 300–1073 K and oxygen concentration range of 20–100%.

Extending the analysis to the complete regime of investigation (Table 2), the variation of $\beta$ as a function of *T* and $X_{O_2}$ is presented in Fig. 6. The surface plot clearly identifies the boundaries for the classical $d_0^2$ law and deviation thereof based on the operating parameters. It can be seen in Fig. 6 that at low T and $X_{O2}$ $\beta$ is less than 2. In a purely diffusion-dominated conversion process, wherein $\beta = 2$, the chemical reactions are extremely fast, and the oxygen is consumed at the particle surface. As T or $X_{O_2}$ is lowered, the reaction rates slow; as a result, the propensity of oxygen to percolate into the particle increases. As this happens, the coefficient $\beta$ reduces to realize values below 2.

### Hypothesis for deviation from $d_0^2$

One of the direct implications of $\beta$ surpassing 2 is that the time time taken for conversion increases. For fixed thermo-physical conditions, the increase in conversion time could only occur if the surface of the particle is starved of reactant. In line with this argument, it is hypothesized that as the heterogeneous reaction rate increases ($C + CO_2 \rightarrow 2CO$), the flux of CO moving radially outward from the surface resists the diffusing $CO_2$ resulting in CO rich and $CO_2$ lean region in the vicinity of the particle surface. The presence of CO rich film results in reduced reactivity of char thereby broadly reducing the conversion process.

**Figure 7:**
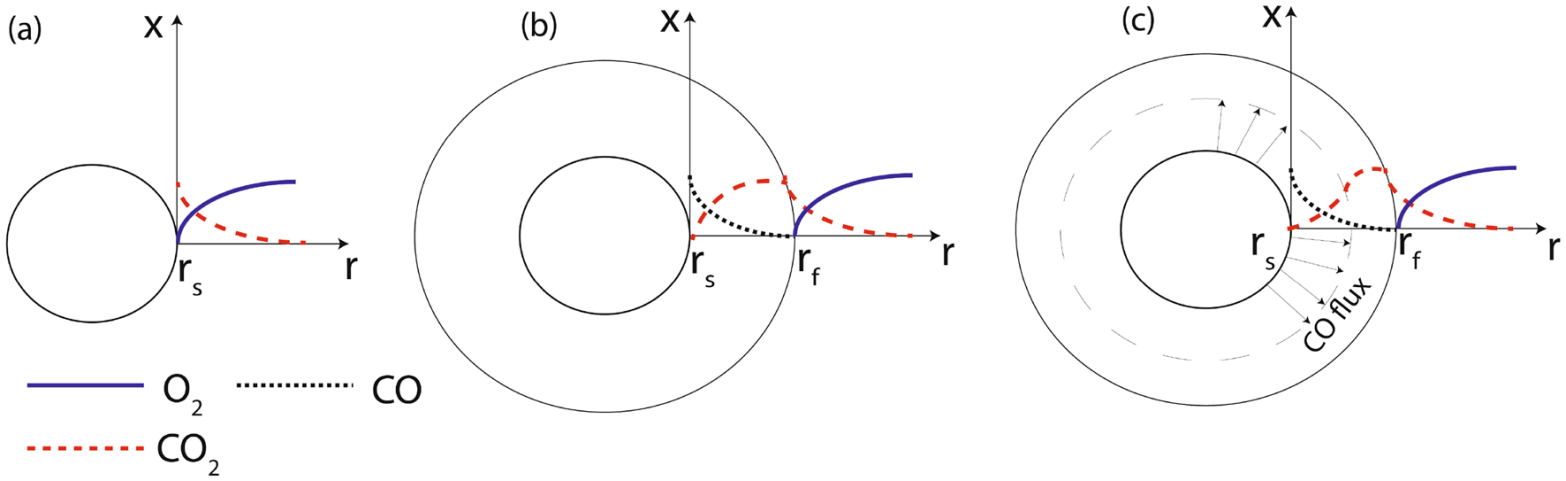
Variation of $O_2$, $CO_2$ and CO mole-fraction in the film (**a**) conventional single-film approach, (**b**) conventional two-film approach (**c**) current approach.

It is important to note that the conventional single film model (Fig. 7a) does not consider the presence of CO film. While the conventional two-film model (Fig. 7b) considers the generation of CO^21,22^, the progressively increasing resistance experienced by $CO_2$ diffusing towards the particle surface due to the CO flux moving in the opposite direction is not accounted for—the $CO_2$ mole fraction approaches zero only at the surface due to reaction with char^23^. This hypothesis is pictorially represented in Fig. 7. The key point sought to be highlighted in the current hypothesis is that the $CO_2$ concentration gradient in Fig. 7c in the vicinity of the particle surface is significantly lower as compared to the conventional approach (when the opposing flux of CO is not considered) (Fig. 7b). In the conventional approach, since the opposing CO flux is not accounted for, a pure diffusion-limited ($d_0^2$) conversion regime gets embedded into the analysis.

In the typical two film model, the hypothesis of CO flux imposing an opposing effect on the $CO_2$ flux is supported by the fact that, while $CO_2$ is generated in a flame sheet surrounded on either side by gaseous media, in so far as CO is concerned, it is bounded on one side by the particle surface. As such, a substantial fraction of the generated CO has to move out in the radial direction due to the combined diffusion and momentum effects. Based on the presented arguments, the presence of a CO film strongly supports the proposed hypothesis.

**Figure 8:**
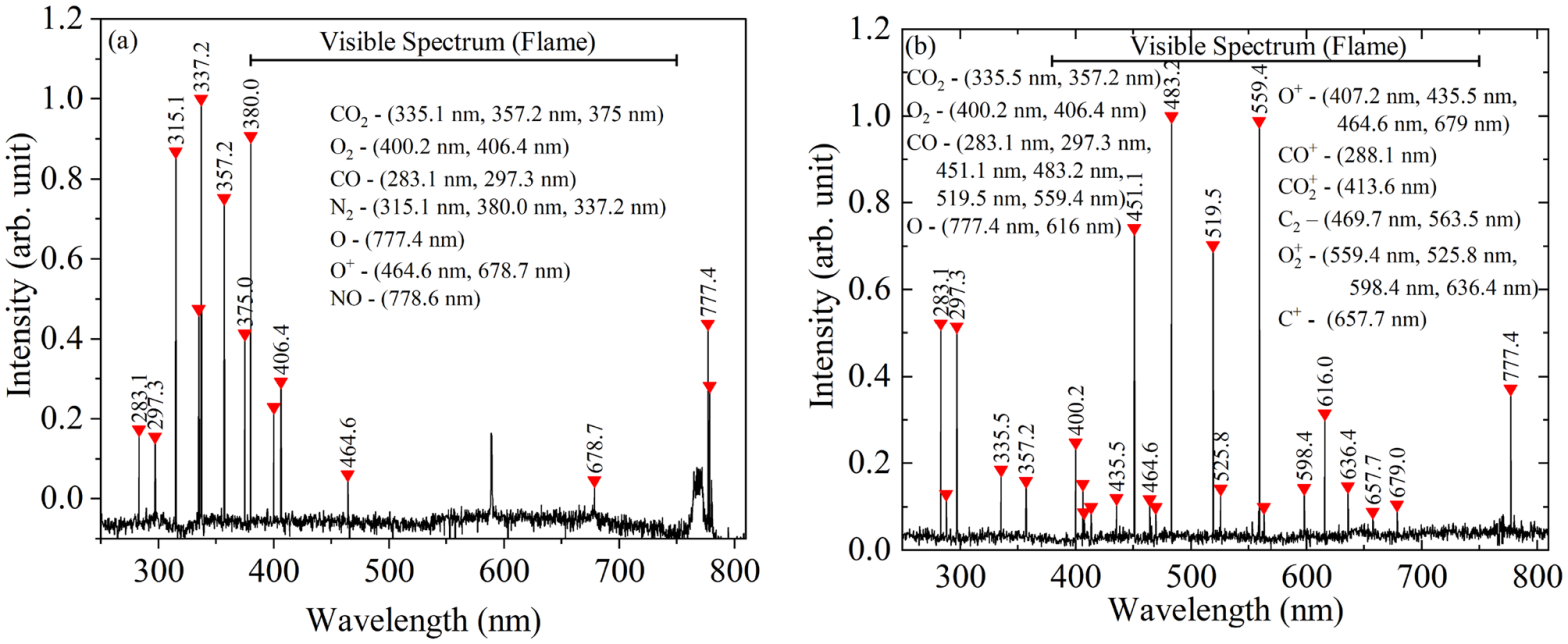
Optical emission spectrum (**a**) No-film mode at 673 K and 20% $O_2$; (**b**) Film mode at 1073 K and 100% $O_2$.

While the presence of a highly luminous film as observed photographically establishes the presence of a reactive media (Fig. 2), spectroscopic investigations clearly establish the presence of a CO film surrounding the particle for cases wherein $\beta$ > 2. The emission spectrum employed to qualitatively characterize the species under (a) no film (single-film) mode identified at 673 K and 20% $O_2$ and (b) CO film (two-film) mode identified at 1073 K and 100% $O_2$, are shown in Fig. 8.

It is observed from Fig. 8 that, along expected lines, under single-film conditions the majority fraction of species comprises of $N_2$ (Normalized intensity ($\lambda _I^*$) − 1) and $CO_2$ ($\lambda _I^*$ − 0.7) followed by $O_2$ ($\lambda _I^*$ − 0.2) and O ($\lambda _I^*$ − 0.4), CO having a minimal $\lambda _I^*$ of 0.16. Additionally, it can be observed that the peaks in the visible region are marginal. In comparison, under two-film mode, significant fraction of CO ($\lambda _I^*$ − 1 − 0.5) contribute to the visible spectrum, visually observed as the film. The $CO_2$ fraction is comparatively less ($\lambda _I^*$ − 0.18) and a large number of ionic and radical species like $O^+$, $CO^+$, $CO_2^+$, $C_2$. $O_2^+$ and $O^+$ are noted. The prevalence of radical and ionic species in two-film model is anticipated owing to the high temperatures generated by CO combustion in the film.

### Evolution of CO film

**Figure 9:**
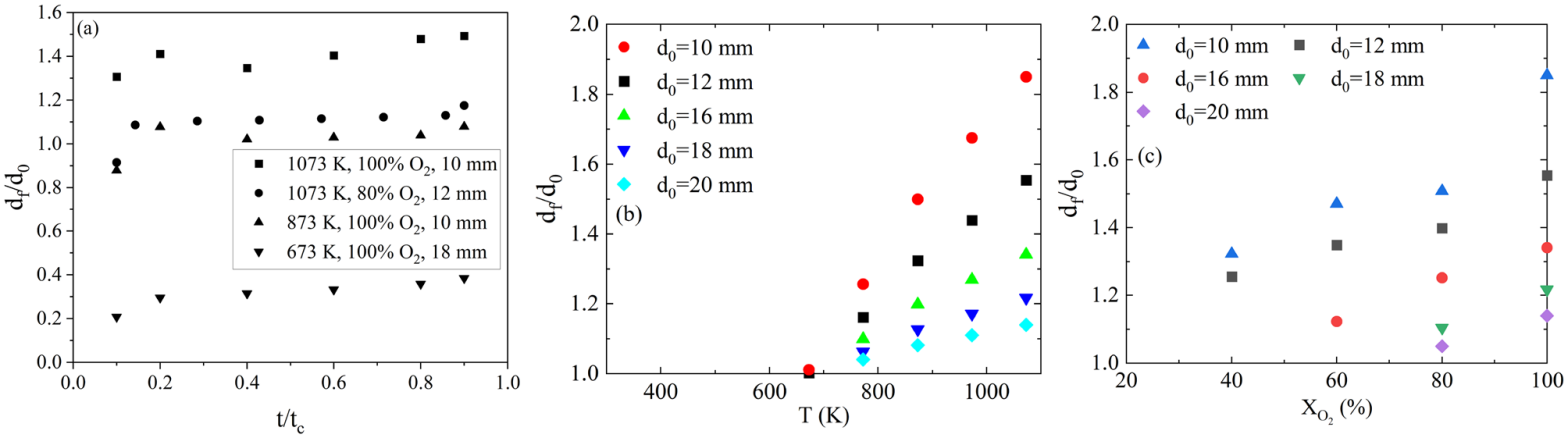
Variation of film diameter $d_f$ to initial particle diameter $d_0$ ratio with (**a**) Normalised conversion time (**b**) temperature (100% $O_2$) and (**c**) $O_2$ concentration (1073 K) (error associated with the determination of $d_f$ is < 0.05 mm).

Extending the influence of CO, experimental observations in the current work suggest that film diameter ($d_f$) is a function of three parameters; $d_p$ (also observed by Shen et al.^24^), T, and $X_{O_2}$. The classical diffusion control approach however assumes that the $d_f$ varies only as function of $X_{O_2}$. The stated dependencies are established based on temporal visual inspections of the evolution of film diameter as indicated in Fig. 9. The evolution of film diameter ($d_f/d_0$) with time, temperature and $X_{O_2}$ across particle sizes is shown in Fig. 9a–c.

It is observed from Fig. 9a that $d_f/d_0$ varies negligibly with time ($\pm 0.05$). The film is established at about 10% conversion (delay owing to the homogeneous mixing and ignition chemistry) and is mapped upto 90% conversion. As such, time averaged film thickness is considered for further analysis.

Figure 9a and b indicates that $d_f/d_0$ increases with an increase in *T* and $X_{O_2}$. It is to be noted that the oxidizer flux is maintained constant (corresponding to Re = 0.1) throughout the experiment and hence does not have any influence on $d_f$. The only plausible explanation for the increase in the film thickness is an increased flux from the particle surface, which is identified as CO from the optical emission spectrum presented in Fig. 8. In summary, the above observations theoretically validate the proposed hypothesis that at higher *T* and $X_{O2}$, the CO flux from the particle increases. Since the diffusion rate of $CO_2$ from the film interface to the particle surface remains the same, a higher CO flux from the particle surface poses enhanced resistance leading to higher conversion time and $\beta > 2$.

### Analytical solution

The hypothesis presented in the previous section finds support in the work by Cornish^25^ investigating heat transfer from concentric spheres. Cornish^25^ report on the reduction of the particle Nusselt number (Nu) with an increase in diameter of the outer sphere. Invoking unity Lewis number approximation, the Prandlt number and Schmidt number are similar, thereby Nu is equivalent to Sherwood number (Sh)^2^. The correction in Sherwood number due to CO film can be formulated as,

3 $$\begin{aligned} Sh_{with\ film}=\frac{h_md_p}{\rho {\mathscr {D}}}\left[ \frac{d_f}{d_f-d_p}\right] =Sh\left[ \frac{d_f}{d_f-d_p}\right] \end{aligned}$$

The mass-loss rate of a single particle burning in a quiescent environment is reported by Annamalai et al.^1^ as,

4 $$\begin{aligned} {\dot{m}}_{p,diffusion}=Sh\pi {\rho }_{g}{\mathscr {D}}d_{p}\mathrm {ln}\mathrm {}(1+B) \end{aligned}$$

In Eq. (4), Sh for a spherical particle is approximated as 2^1^, gas density (${\rho }_g$) is obtained from the equation of state and $\mathscr {D}$ is the multi-component mass diffusion coefficient estimated using kinetic theory-based Chapman-Enskog equation. The transfer number B is ratio of stochiometric coefficients. Equating Eq. (4) to the temporal derivative of particle mass and integrating with respect to *d* and *t* with initial condition (at $t=0,\ d=d_0$) and terminal condition ( $at\ t\rightarrow t_c,\ d\rightarrow 0$) yields the classical $d_0^2$ correlation,

5 $$\begin{aligned} t_c=\frac{d^2_0}{\alpha }; \, \,\alpha =\frac{6Sh{\rho }_g\mathscr {D}{\mathrm {ln} \left( 1+B\right) \ }}{{\rho }_p} \end{aligned}$$

For a particle within a CO film, Sh in Eq. (4) is corrected with Sh defined in Eq. (3) as,

6 $$\begin{aligned} {\dot{m}}_{p,diffusion}=Sh\pi {\rho }_g\mathscr {D}d_p{\mathrm {ln} \left( 1+B\right) \ }\left[ \frac{d_f}{d_f-d_p}\right] \end{aligned}$$

Equating Eq. (5) to the temporal evolution of mass,

7 $$\begin{aligned} -\frac{{\rho }_p\pi d^2_p}{6}\frac{d}{dt}\left( d_p\right) =Sh\pi {\rho }_g\mathscr {D}d_p\mathrm {ln}\mathrm {}(1+B)\ \left[ \frac{d_f}{d_f-d_p}\right] \end{aligned}$$

Integrating Eq. (6) and applying the initial and terminal conditions as in the case of Eq. (4), the modified $d_0^2$ law is obtained as follows,

8 $$\begin{aligned} t_c=\frac{d^2_{0,p}}{{\alpha '}}\left[ 1-\frac{2d_{0,p}}{3d_f}\right] \end{aligned}$$

$$\begin{aligned} {\alpha '}=\frac{3Sh{\rho }_g\mathscr {D}{\mathrm {ln} \left( 1+B\right) \ }}{{\rho }_p} \end{aligned}$$

It is observed from Eq. (7) that, in presence of a CO film, the conversion time in addition to being a function of $d^2_{0,p}$ also depends on the ratio of particle diameter to film diameter $\frac{d_{0,p}}{d_f}$, thereby deviating from the classical $d_0^2$ regime.

#### Limiting conditions

The analytical correlation, Eq. (8), can be posed with three limiting conditions with respect to the particle diameter to film diameter ratio, $R = 2d_{0,p}/3d_f$.

*Case 1:* $\mathbf {R=1}\rightarrow$ Considering $d_{0,p} = 20$ mm as an example, to obtain R=1; $d_f = 13.3$ mm. This case is not possible as the CO film forms at the exterior of the particle and $d_f$ is always greater than $d_p$.

*Case 2:* $\mathbf {R \gg 1}$ $\rightarrow$ It is seen in Case 1 that for $R=1$, $d_f<d_p$. Thereby, for $R \gg 1$, it concurs $d_f \ll d_p$. Since $d_f$ is always greater than $d_p$, Case 2 is impossible .

*Case 3:* $\mathbf { R \ll 1}$ $\rightarrow$ This case is a possible when the film diameter is a magnitude larger than the particle diameter, $d_f \gg d_p$. Under such circumstances, the extremely large film acts as an infinite ambient medium, similar to the single-film approach, and Eq. (8) reduces to, $t_c=(d_{0,p}^2)/ \alpha ^{'}_{diffusion}$.

Conclusively, depending on the prevailing thermophysical conditions, $R<1$ and under limiting condition of $R \ll 1$, Eq. (8) reduces to the conventional $d_0^2-law$.

### Validation of hypothesis

The analytical correlation (Eq. 8) obtained for a single particle conversion time as a function of $d_0$ and $d_f$ is a typical initial value problem. The solution requires knowledge of $d_0$, $d_f$ and ${\alpha '}$. The transfer number B, under a two-film consideration, is formulated by Turns^17^ as,

9 $$\begin{aligned} B=\frac{2Y_{O_{2,\infty }}-\left[ \frac{{\vartheta }_s-1}{{\vartheta }_s}\right] Y_{CO_2,s}}{{\vartheta }_s-1+\left[ \frac{{\vartheta }_s-1}{{\vartheta }_s}\right] Y_{CO_2,s}} \end{aligned}$$

In Eq. (9), ${\vartheta }_s$ is the stoichiometric ratio for C-CO${}_{2}$ reaction at the particle surface and $Y_{O_{2,\infty }}$ is the mass fraction of oxygen at infinity. $Y_{CO_2,s}$ is the mass fraction of CO${}_{2}$ at the surface obtained iteratively with an initial guess of surface temperature and considering that under a purely diffusion-limited regime $Y_{CO_2,s}\rightarrow 0.$ The transfer number thus obtained from Eq. (9) is used to arrive at the ratio of particle diameter to film diameter employing the analytical correlation obtained from solving energy and specie balance at the particle surface and the CO film (flame)^17^,

10 $$\begin{aligned} \frac{d_f}{d_{p,0}}=\frac{\mathrm {ln}\mathrm {}(1+B)}{{\mathrm {ln} \left( \frac{{\vartheta }_s-1}{{\vartheta }_s}\right) \ }} \end{aligned}$$

The flame diameter d${}_{f}$ obtained using the analytical correlation (Eq. 10) is compared with the experimental film diameter recorded photographically in the current work. The comparison is consolidated in Table 4. With the knowledge of ${d_0}_{.p}$, $\frac{d_f}{d_{p,0}}$, and ${\alpha '}$ the total conversion time under different thermophysical conditions is estimated using Eq. (8) and compared with experimental burn times from the current work. The analysis is consolidated in Table 5.

**Table 5:** Comparison of the film diameter and the total burn time estimated by the analytical correlations and experiments.

T(K)	$X_{O_2} (\%)$	B	$\alpha$	$d_f$	$\sim d_f (\%)$	$t_c$		$\sim t_c (\%)$
						Corr.	Exp.	
1073	100	0.74	4.1E-06	$20.23^\# (22.93^*)$	–13.3	$14.73^a (19.04^e)$	19.1	23.0 (0.5)
	80	0.59	1.4E-06	$16.84^\# (19.26^*)$	–14.3	$46.39^a (50.2^e)$	45.0	–3.1 (–11.6)
	60	0.44	8.9E-07	$15.41^\# (15.23^*)$	1.1	$69.99^a (69.37^e)$	58.8	–19.1 (–18.0)
	40	0.3	4.1E-07	$12.7^\# (10.75^*)$	15.4	$126.91^a (101.41^e)$	104.5	–21.4 (3.0)
873	100	0.74	2.1E-06	$19.75^\# (22.93^*)$	–16.1	$34.57^a (37.01^e)$	36.0	4.0 (–2.8)
	80	0.59	1.3E-06	$15.87^\# (19.26^*)$	–21.3	$41.79^a (57.56^e)$	49.2	15.0 (17.0)
	60	0.44	5.8E-07	$12.8^\# (15.23^*)$	–19.0	$91.82^a (107.75^e)$	86.8	–5.7 (–24.1)
	40	0.3	2.8E-07	$11.37^\# (10.75^*)$	5.5	$160.97^a (147.75^e)$	135.7	–18.7 (–8.9)
673	100	0.74	1.6E-06	$19.12^\# (22.93^*)$	–19.9	$43.54^a (47.41^e)$	46.8	7.0 (–1.3)
	80	0.59	9.0E-07	$15.43^\# (19.26^*)$	–24.8	$69.19^a (79.65^e)$	75.5	8.4 (–5.5)

$^{\#}$ Experimental data; $^{*}$ Analytical estimate; $^{a}$ Burn time estimated using analytically computed film diameter; $^{e}$ Burn time estimated using experimentally estimated film diameter; $\sim d_f$ deviation in experimental and analytical film diameter; $\sim t_c$ deviation in experimental and theoretical burn times.

Reviewing the data consolidated in Table 5, it can be observed that, the flame diameter and the total conversion time estimated from the analytical approach remain very close to the experimental observations with the maximum difference in both the cases being within 25%.

## Error quantification and propagation

The experimental setup and methodology introduce errors in the final results. The errors are introduced from (1) the experimental setup in terms of uncertainties in the measured value, (2) the methodology and procedure followed during the experiment and (3) the propagation of error from its introduction to the final result.

With regard to the experimental setup, Table 6 consolidates the error associated with the various measurements made in the experiments. To address the errors arising from methodology, each data point was repeated at least five times, and the the standard deviation was found to be less than 5%.

**Table 6:** Errors associated with experimental measurements.

Measured property	± deviation
Temperature ($^0$C)	0.1
$O_2$ concentration (%)	0.01
$O_2$ flowrate (lpm)	0.05
Particle mass (mg)	0.1
Initial particle size (mm)	0.2
Video-graphic particle size measurement (mm)	0.05

The errors from the experimental setup and the procedure are propagated to the final calculated values based on the standard approaches detailed by Clifford^26^.

## Conclusions

The current work has experimentally and analytically addressed combustion of biomass char in oxygen enriched and thermally supported environments with specific emphasis on conditions conventionally not addressed. Spherical char particles sized between 8 and 20 mm are subjected to temperatures (T) in range of 300 K to 1073 K and $O_2$ mole fraction ($X_{O_2}$) in the range of 20% to 100%. Following are the key observations;

- While the conversion process follows the classical $d_0^2$-law under certain low T- $X_{O_2}$ conditions, at high T- $X_{O_2}$ deviation from the classical $d_0^2$-law is observed with the power of $d_0$ surpassing 2, a first of its kind observation.
- A surface plot is presented identifying 673 K and 40% $O_2$ as transition conditions for deviation from $d_0^2$-law; single-film to two-film transition.
- It is hypothesized that a convective flux of CO film from the particle surface curtails $CO_2$ diffusion towards the surface resulting in significantly reduced $CO_2$ concentration near the surface of the particle. The CO film identified and characterized by image processing and emission spectroscopy techniques is found to vary as a function of three parameters—$d_0$, T and $X_{O_2}$ while conventionally, only functional dependence on $X_{O_2}$ is considered.
- The proposed hypothesis is analytically formulated by amending the Sherwood number and solving for mass-loss rate of the particle to arrive at conversion time ($t_c$)- $d_0$ correlation. It is found that under conditions wherein CO film prevails the $t_c$ in addition to being a function of $d_0^2$, is also a function of ratio of $d_0$ to film size $d_f$.
- The hypothesis is validated using experimental observations and agrees within 10% deviation for most cases and less than 25% deviation in all cases.

## Data Availability

The data that support the findings of this study are available from the corresponding author upon reasonable request.

## References

[1] Annamalai K, Ryan W (1993). Interactive processes in gasification and combustion-ii. Isolated carbon, coal and porous char particles. Prog. Energy Combust. Sci..

[2] Law CK (2010). Combustion Physics.

[3] Dasappa, S., Sridhar, H., Paul, P., Mukunda, H. & Shrinivasa, U. On the combustion of wood-char spheres in o2/n2mixtures-experiments and analysis. In *Symposium (International) on Combustion*, vol. 25, 569–576 (Elsevier, 1994). 10.1016/S0082-0784(06)80687-9.

[4] Mukunda, H., Paul, P., Srinivasa, U. & Rajan, N. Combustion of wooden spheres-experiments and model analysis. In *Symposium (International) on Combustion*, vol. 20, 1619–1628 (Elsevier, 1985). 10.1016/S0082-0784(85)80657-3.

[5] Annamalai K, Puri IK (2006). Combustion Science and Engineering.

[6] Haugen NEL, Tilghman MB, Mitchell RE (2014). The conversion mode of a porous carbon particle during oxidation and gasification. Combust. Flame.

[7] Kreitzberg T, Phounglamcheik A, Haugen NEL, Kneer R, Umeki K (2022). A shortcut method to predict particle size changes during char combustion and gasification under regime ii conditions. Combust. Sci. Technol..

[8] Makino A (2014). An attempt for applying formulation of the carbon combustion in the stagnation flowfield to some experimental comparisons related to the boundary layer combustion. Combust. Flame.

[9] Tsuji H, Matsui K (1976). Aerothermochemical analysis of combustion of carbon in the stagnation flow. Combust. Flame.

[10] Matsui K, Kôyama A, Uehara K (1975). Fluid-mechanical effects on the combustion rate of solid carbon. Combust. Flame.

[11] MATALON M (1981). Weak burning and gas-phase ignition about a carbon particle in an oxidizing atmosphere. Combust. Sci. Technol..

[12] Waibel, R. T. & Essenhigh, R. H. Combustion of thermoplastic polymer particles in various oxygen atmospheres: Comparison of theory and experiment. In *Symposium (International) on Combustion*. *Fourteenth Symposium (International) on Combustion,* vol. 14, 1413–1420. 10.1016/S0082-0784(73)80126-2 (1973).

[13] Makino, A. & Law, C. K. Quasi-steady and transient combustion of a carbon particle: Theory and experimental comparisons. In *Symposium (International) on Combustion*, vol. 21, 183–191. 10.1016/S0082-0784(88)80245-5.

[14] Qi M (2022). System perspective on cleaner technologies for renewable methane production and utilisation towards carbon neutrality: Principles, techno-economics, and carbon footprints. Fuel.

[15] Ye C, Wang Q, Yu L, Luo Z, Cen K (2018). Characteristics of coal partial gasification experiments on a circulating fluidized bed reactor under co2o2 atmosphere. Appl. Therm. Eng..

[16] Cerone N (2020). Experimental investigation of syngas composition variation along updraft fixed bed gasifier. Energy Convers. Manag..

[17] Turns SR (1996). Introduction to Combustion.

[18] Patel V, Shah R (2018). Experimental investigation on flame appearance and emission characteristics of lpg inverse diffusion flame with swirl. Appl. Therm. Eng..

[19] Avedesian, M. & Davidson, J. Combustion of carbon particles in a fluidised bed. *Trans. Inst. Chem. Eng.* **51**, 121 (1973). https://www.icheme.org/knowledge-hub/download/75009?filename=51ap0121.pdf.

[20] Annamalaim K, Caton J (1987). Distinctive burning characteristics of carbon particles. Can. J. Chem. Eng..

[21] Zhang Z (2022). Numerical study on heterogeneous reaction characteristics of a single coal char particle under air-and oxy-fuel combustion: Effects of particle motion. Fuel.

[22] Zhang Z (2021). Reaction behaviors of a single coal char particle affected by oxygen and steam under oxy-fuel combustion. Fuel.

[23] Lewtak R, Milewska A (2013). Application of different diffusion approaches in oxy-fuel combustion of single coal char particles. Fuel.

[24] Shen Z (2018). In situ experimental and modeling study on coal char combustion for coarse particle with effect of gasification in air (o2/n2) and o2/co2 atmospheres. Fuel.

[25] Cornish, A. R. H. Note on minimum possible rate of heat transfer from a sphere when other spheres are adjacent to it. *Trans. Inst. Chem. Eng.* **43**, T332–T333 (1965). https://www.icheme.org/knowledge-hub/download/74729?filename=43ap0332.pdf.

[26] Cliford, A. Multivariate error analysis: A handbook of error propagation and calculation in many-parameter systems (1973).

